# COVID-19-Related Intolerance of Uncertainty and Mental Health among Back-To-School Students in Wuhan: The Moderation Effect of Social Support

**DOI:** 10.3390/ijerph18030981

**Published:** 2021-01-22

**Authors:** Lijun Zhuo, Qian Wu, Hong Le, Hao Li, Ling Zheng, Guoqing Ma, Hongbing Tao

**Affiliations:** School of Medicine and Health Management of Tongji Medical College, Huazhong University of Science and Technology, Wuhan 430030, China; lijunzhuo@hust.edu.cn (L.Z.); hustayla7@hust.edu.cn (Q.W.); lehong@hust.edu.cn (H.L.); leohao@hust.edu.cn (H.L.); zhengling@hust.edu.cn (L.Z.); mgq@hust.edu.cn (G.M.)

**Keywords:** intolerance of uncertainty, social support, mental health, college students, COVID-19

## Abstract

The current wave and future trend of the novel coronavirus disease 2019 (COVID-19) has triggered public uncertainty, causing unbearable psychological pressure on people. A cross-sectional online questionnaire was conducted among back-to-school students in Wuhan from 31 August 2020, to 14 September 2020, by using convenience sampling. A total of 1017 participants voluntarily provided sociodemographic characteristics and accomplished the following scales: the Intolerance of Uncertainty Scale (IUS-12), the Social Support Scale (SSQ), the Generalized Anxiety Disorder Scale (GAD-7), the Patient Health Questionnaire-9 (PHQ-9), and the Insomnia Severity Index-7 (ISI-7). Results revealed that the levels of anxiety, depression, and insomnia were moderate, moderate and subthreshold, respectively. A one-way multivariate analysis of variance indicated that those with different attitudes toward the trajectory of the COVID-19 epidemic in China showed significantly different results in anxiety and depression (*p < 0.001*). Moderation modeling implicated that social support significantly moderated the predictive relationship between intolerance of uncertainty and mental health variables including anxiety and depression, but failed on insomnia. Findings indicate that back-to-school students in Wuhan experience mental health problems and improving social support measures could buffer the effect of intolerance of uncertainty with respect to COVID-19 on mental health.

## 1. Introduction

Coronavirus Disease 2019 (COVID-19), as an infectious disease caused by severe acute respiratory syndrome, gained a foothold worldwide rapidly, and was declared a “public health emergency of international concern” by the World Health Organization [1,2]. National and local strategies such as keeping social distance, quarantine, school closures, have been implemented to respond to the novel pandemic disease. Wuhan was the epicenter of the COVID-19 epidemic, which began in December 2019 and continued to be well controlled until April 2020. Previous studies indicated that public health problems in the post-crisis era were serious and correlational researches should be carried out actively, especially on public mental health problems [3,4]. Because the COVID-19 pandemic is unprecedented and akin to “navigate the uncharted”, mapping the genome of the flu strain and developing vaccines are long progress [2]. At present, the spread of the national and local epidemic is curbed, but the risk of sporadic cases and local partial outbreaks remains. Moreover, recurrences in cured patients had been reported in some countries. In September 2020, amid the rising cases of infection worldwide, college students in Wuhan returned to school, causing increased population mobility and the high risk of COVID-19 pandemic rebound. Therefore, the future trend of the COVID-19 pandemic remains uncertain and the pandemic is likely to spike again. The resulting COVID-19-induced uncertainty has an influence on every aspect of our lives, including our mental health [5].

### 1.1. Public Crisis and Mental Health

Previous cross-sectional and longitudinal studies implicated that public health crisis triggered mental health problems among the public, even in post-crisis periods. A systematic review suggested that public mental health problems, including stress, depression, and anxiety, were more severe during the severe acute respiratory syndrome (SARS )outbreak and lasted until 4 years later in the post-SARS period without reasonable interventions [3]. In Taiwan, China, psychological distress associated with demographic factors among residents during a post-SARS crisis was identified by investigating 1278 samples, aged 18 and above [6]. Similarly, college students in Guangzhou, China had hemagglutinin type-1 neuraminidase type-1 influenza virus(H1N1) related mental distress which was associated with cognitive variables [7]. Recent studies emphasized mental health problems including anxiety, depression, insomnia, and distress among children, adolescents, health care workers, discharged patients, and college students who were isolated at home in China during the COVID-19 pandemic [8,9,10,11].

The mental health of back-to-school students in the post-COVID-19 era has not yet been explored. The current COVID-19 pandemic, with the 2003 SARS epidemic, and other past epidemics have shown that, public health crisis had a long-term impact on people’s psychological health, hence, the impact factors should be explored actively [12]. Moreover, the severity of the COVID-19 far exceeds the severity of the SARS pandemic and the H1N1 influenza pandemic. Therefore, understanding influencing factors of mental health during and following the COVID-19 pandemic is necessary [13]. Post-traumatic stress and poor mental health are more likely to cause suicidal behavior, especially for female college students [14]. Therefore, investigating the mental health problems of college students and taking effective intervention measures are essential.

### 1.2. Intolerance of Uncertainty and Mental Health

Intolerance of uncertainty (IU) was first defined by Freeston as a reaction that includes cognition, emotion, and behavior towards uncertain events [15]. In 2016, Carleton defined the IU as the incapacity to deal with uncertain situations or unpredictable events [16]. IU was regarded as an important factor to influence public mental health [17,18]. Numerous researches showed that generalized anxiety, depression, or other mental health problems were significantly associated with IU among a broad range of samples [16], such as mothers of children with autism spectrum disorder [19], public safety personnel [20], women diagnosed with ovarian cancer [21], pregnant women [22], and men on active surveillance for prostate cancer [23]. Furthermore, a high level of IU scores implicated serious psychological problems [16]. More mediation effects and fewer moderation effects were explored in prior researches to mediate or moderate the association between IU and mental health. The coping strategy, rumination and fear of COVID-19 as mediators could mediate the relationship between IU and mental wellbeing, whereas, loneliness as a moderator could moderate the relationship between IU and mental health [19,21,24].

People have been more concerned about their mental health than their physical health during the COVID-19 pandemic [25], and negative COVID-19-related mental issues will be lasting and profound because the outcome of the pandemic is uncertain [26]. In addition, the continuous confirmed cases, inadequate information, and negative impact on the multi-aspect life had caused great uncertainty, which consequently increases anxiety [27]. The relationship between IU and mental health had been explored in previous studies among different groups of people. However, the association between IU and negative mental health among college students returning to Wuhan has not been conducted in recent research.

### 1.3. Social Support and Mental Health

Previous research has proven that social networks among individuals play an essential role in treating psychological problems [28,29,30]. The excessively negative sentiment aroused by unpredictable catastrophes would be a sustaining mental disorder if no appropriate interventions are made [31]. Social support, as a form of mutual communication and connection network, could be divided into emotional support, instrumental support, and informational support [32]. Such support was robustly associated with mental health among different samples, such as the aging population [33], college students [34], and health care workers [35], in several cross-sectional studies and longitudinal studies. Moreover, the relationship between social support as an independent variable or a moderator and mental health was explored in previous studies. Regression was used to analyze the relevant relationship between social support and mental health of 1242 university students. The results implicated a significant positive relationship, university students had better mental health to sustain themselves against crises and stress when they obtain strong social support from their friends, family, and teachers [34]. In a study among 632 undergraduate students, social support was confirmed to moderate the relationship between stress and depression [36]. Social support was also applied to explore the moderation effect between internalized stigmatization and mental health of people who worked or studied in Hubei province before the COVID-19 outbreak and finding revealed that mental health problems could be buffered by high levels of social support [37].

A cross-sectional study proposed that social support moderated the relationship between IU and mental health among women diagnosed with ovarian cancer [21]. However, no extant research has explored social support as a moderator to buffer the association between IU and mental health among college students in the post-COVID-19 period in China. The current study aims to explore the relationship between IU and mental health, and whether the relationship can be moderated by social support. Based on literature review, this study proposes the following hypotheses:

**Hypothesis** **1** **(H1).** *IU would be positively associated with poor mental health, whereas social support would be negatively linked to poor mental health.*

**Hypothesis** **2** **(H2).** *Social support would moderate the relationship between IU and mental health.*

## 2. Materials and Methods

### 2.1. Design and Procedure

This cross-sectional study used an online questionnaire to measure mental health variables, IU, and social support of back-to-school students in Wuhan by the ‘Questionnaire Star Application’. Participants could click the study link or scan the QR code to answer the questionnaire items independently after they submit their informed consent. In addition, this study met ethical protocols (No: IORG0003571).

Convenience sampling was used to collect maximized samples, and several remedies were used to reduce convenient sampling bias [38]. First, respondent anonymity and the confidentiality of personal information were guaranteed. Second, paginations between demographic information and measurement scales were set to create a temporal separation. Third, The IU scale and social support scale were set in the first and final parts, respectively, to attempt to control psychological retrieval cues.

Data were collected online from 31 August 2020, to 14 September 2020. The sample size was estimated on the basis of 10 to 20 times the number of questionnaire items [39]. The study had 47 items, and the sample size was 940 at least. Of the 1332 survey participants, 1017 returned valid responses, and the effectivity rate was 76.35%.

### 2.2. Participants

Back-to-school college students from different universities in Wuhan were invited to participate in our online study. The inclusion criteria of participants were: (i) voluntarily participates in this research; (ii) a student in Wuhan before the COVID-19 outbreak and currently; (iii) and at least 18 years old. The exclusion standards were as follows. First participants who only completed one or two parts of the scales were excluded. Second, samples without answers to key items cannot be computed; hence, they were also excluded.

### 2.3. Measures

#### 2.3.1. Intolerance of Uncertainty

IU was measured using the Chinese version of the Intolerance of Uncertainty Scale-Short Form (IUS-12). The scale has a stable two-factor structure, including prospective anxiety and inhibitory anxiety, for example, “unforeseen events upset me greatly” and “uncertainty keeps me from living a full life” [40]. A 5-point Likert-type scale (1 = “not at all characteristic of me;” 2 = “a little characteristic of me;” 3 = “somewhat characteristic of me;” 4 = “very characteristic of me;” 5 = “entirely characteristic of me”) was applied to this scale to measure the IU of participants. The possible scores of this scale ranged from 12 to 60. A higher score means that the IU is at a high level. The current study’s internal consistency was excellent (α = 0.849).

#### 2.3.2. Social Support

Social support was measured by a short form of the Chinese version of the Social Support Questionnaire (SSQ). The questionnaire has six-items, such as “you can easily find someone that you really count on to distract you from your worries when you feel under stress.” This scale is highly correlated with 27-items SSQ, and its internal reliability is excellent (α = 0.90) [41]. A 7-point Likert-type scale (from 1 = “strongly disagree;” to 7 = “strongly agree”) was used in this scale. The current study’s internal consistency was excellent (α = 0.962).

#### 2.3.3. Anxiety

Anxiety was measured by a short form of the Chinese version of the seven-item General Anxiety Disorder-7 (GAD-7), developed by Andrea et al. [42]. The scale includes “I feel tense or “wound up” “I get a sort of frightened feeling as if something awful is about to happen” among others. A 4-point scale was used (1 = “not at all” to 4 = “nearly every day”). The current study’s internal consistency is excellent (α = 0.929).

#### 2.3.4. Depression

Depression was measured by a short form of the Chinese version of the nine-item Patient Health Questionnaire-9 (PHQ-9), developed by Kroenke et al. [43]. The scale includes “little interest or pleasure in doing things;” and “feeling down, depressed, or hopeless” among others. A 4-point scale was used (1 = “not at all;” to 4 = “nearly every day”). The current study’s internal consistency was excellent (α = 0.918).

#### 2.3.5. Insomnia

Insomnia was measured by a short form of the Chinese version of the seven-item Insomnia Severity Index-7 (ISI-7) [44]. The scale includes “difficulty falling asleep;” “difficulty staying asleep” among others. A 5-point scale was used (1 = “none;” to 5 = “very severe”). The current study’s internal consistency was excellent (α = 0.903).

### 2.4. Data Analysis

Data were analyzed using SPSS Version 24 (IBM Corp., Armonk, NY, USA). First, the descriptive variables were expressed in terms of quantity and frequency or mean and standard deviation. Pearson’s correlations were performed to test the correlation among IU, social support, and mental health variables.

Second, Levene’s test was used to analyze the homogeneity of variances. The test showed the following results: *p* = 0.890, 0.762, 0.334, *p >* 0.05, respectively. Therefore, one-way multivariate analysis of variance was applied to assess the differences in anxiety, depression, and insomnia among three groups, which had been classified by their attitude to the COVID-19 epidemic in China whether rebound.

Finally, moderate modeling in PROCESS macro for SPSS was applied to analyze whether IU and social support predicated the mental health variables and whether social support moderated the relationship between IU and mental health variables. All variables in this study had been standardized. The effects of sex, age, education level, department category, and the time excepted to graduate as covariates were controlled by moderate modeling.

## 3. Results

### 3.1. Summary of Measured Variables

Table 1 shows the demographics information of participants. Table 2 shows the mean ± SD of IU, anxiety, depression, insomnia and social support were 36.01 ± 7.379, 11.36 ± 4.297, 14.18 ± 5.180, 13.76 ± 5.495, and 29.18 ± 9.521, respectively. Pearson’s correlations analyses indicate that all variables were significantly correlated with one another (*p <* 0.001). A significant and positive correlation existed between IU and mental health variables, and a negative correlation existed between social support and other variables.

### 3.2. Comparison between the Mental Health among Different Groups

Table 3 shows that the differences in anxiety scores and depression scores among the three groups were statistically significant (F = 7.721, *p <* 0.001; F = 4.868, *p* = 0.008). By contrast, differences in insomnia scores among three groups are not statistically significant (F = 2.855, *p =* 0.058). Additionally, the mean anxiety and depression scores of group 1 and group 3 were at a higher level than the scores of group 2.

### 3.3. Moderation Effect of Social Support between IU and Mental Health

Table 4 suggests that IU positively predicted anxiety, depression and insomnia (β = 0.40, 0.39, and 0.34, respectively, *p* < 0.001). The interaction effect between IU and social support on anxiety and depression was significantly negative (β = −0.07 and −0.06, respectively, *p <* 0.001), whereas, the results of the interaction between IU and social support on insomnia were not significant (β = −0.04, *p* >0.05).

**Figure 1:**
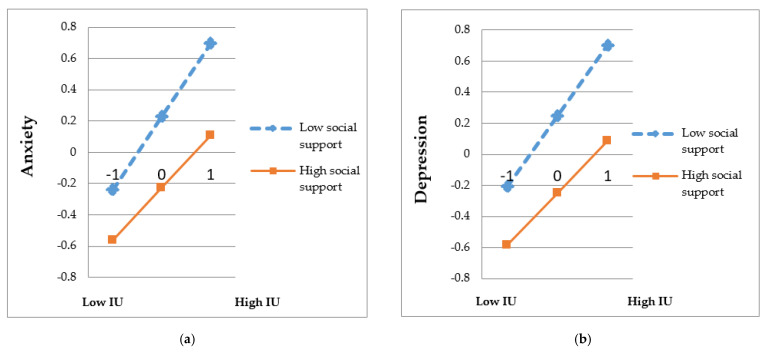
Moderation effect of social support between IU and mental health variables. IU, intolerance of uncertainty (**a**) Description of moderation effect of social support between IU and anxiety. (**b**) Description of moderation effect of social support between IU and depression.

Table 5 shows the moderating effect of social support between IU and mental health outcomes, including anxiety and depression (*p* < 0.001). The results shed further light on the effects of IU on anxiety and depression were significantly weaker at higher levels of social support (β = 0.33 and 0.33, *p <* 0.001) than that at low levels of social support (β = 0.47 and 0.45, *p <* 0.001). Simple effects of moderation are depicted in Figure 1. The slope of low support was steeper than that of high social support in anxiety and depression. This result indicated that at a level of low social support, college students with high IU scores were associated significantly with much more anxiety and depression outcomes, which could be weakened by high social support.

## 4. Discussion

The COVID-19 pandemic, as an unprecedented public health crisis, has resulted in profound mental health problems, especially regarding its uncertain future trend [10]. Highlighted mortality cases and confirmed cases, disrupted routine and plans, and various news aggravate COVID-19-related uncertainty [12,45]. The present study aims to explore whether IU and social support affect mental health outcomes (anxiety, depression and insomnia), and whether social support buffers the relationship between IU and mental health among back-to-school students in Wuhan during the COVID-19 pandemic, during which treatment and future development trends are uncertain.

The IU score was 36.01 ± 7.379, which is similar to that of Turkish samples during the novel COVID-19 pandemic period [24], which is seriously higher than that of adult individual samples in Mexico during the H1N1 pandemic in 2009 [46]. Mental health problems are also much more severe than those during the SARS [6] and H1N1 crisis [7]; hence, they should be given more attention. Anxiety and depression scores were all moderate according to the categories established in the previous literature [9], which are higher than the scores of adult samples who lived in the United Kingdom during the COVID-19 pandemic [26]. College students returning to Wuhan, the first epicenter of the infection, may have faced different living environments, school closures, stigma, and misinformation boom [8,45]. Insomnia score was at the subthreshold level according to the categories established in the previous literature [9], reflecting that the COVID-19 pandemic has little effect on the insomnia of college students.

The emergence of IU as a significant predictor of mental health was positively associated with mental health outcomes, which were quadrated with previous analysis conclusions [24]. Fear of the unknown and excessive worry always result in severe self-pressure and loneliness, which trigger physical sensations related to anxiety, depression and insomnia, especially in the context of the COVID-19 pandemic [21,40]. Uncertainty about the outcomes of COVID-19 worldwide is one of the important manifestations of uncertainty. Our findings implicated that college students with different attitudes were closely linked to their mental health, including anxiety and depression. College students who are certain or uncertain about the resurgence of the COVID-19 epidemic in China have greater anxiety and depression than college students who hold the opposite opinion. This result indirectly reveals that COVID-19-related uncertainty has a deeply destructive impact on mental health. Moreover, this finding is in line with previous results that found that fear of COVID-19 pandemic has a malign influence on mental wellbeing in Turkish individuals [24]. In addition, the pandemic in China is at risk of rebound. Therefore, mental health would have worsened if reasonable interventions had not been taken [3]. The cognitive behavioral interventions aimed at IU may be able to mitigate the risk of mental illness under times of uncertainty [47].

Social support was negatively related to adverse mental health and could buffer the relationship between IU and mental health outcomes (anxiety and depression) as well. The result is lined with the “buffer” hypothesis, which social support as the buffering effect can moderate the mental health problems [48]. This finding further implicates that high social support can weaken the impact of IU on anxiety and depression. Social support from family, teachers and peers could eliminate loneliness and improve self-concept to some extent, further decreasing anxiety and depression [30]. Previous researches concentrated on the moderation effect between IU and mental health including coping strategy, and rumination [24,26], which are internal elements of individuals and could not be improved in the short term. By contrast, social support could be provided by external elements. First, private and convenient psychological counseling rooms or online channels could provide timely and professional psychological assistance to those students in need. Therefore, related departments should pay attention to college students’ mental health changes and carry out mental health education regularly. Last but not least, individuals should forwardly strengthen their communication with immediate people to establish good social relations. Anxiety and depression will result in various severe problems; low academic achievement and high absenteeism rate were frequent occurrences among persistently anxious and depressed students unlike non-anxious, non-depressed students [49]. Therefore, our findings provide targeted interventions to improve the mental health of college students. Evidence-based treatments should be considered in relieving mental health symptoms including anxiety and depression.

Our research failed to prove social support as a buffering effect to moderate the relationship between IU and insomnia at a test level of 0.05. This result is akin to previous research conclusion that social support cannot moderate the association between the sleep reactivity caused by increasing risk of incident and insomnia, which may be interpreted by the internal mechanism and nature of insomnia [50]. Previous studies showed that insomnia phenotypes were related to genetic factors, which could explain approximately 30–40% and also indicated that insomnia as a symptom of psychopathology was related to emotional disorders and mood status, such as depression, and anxiety [51,52]. Further research showed that social support could affect insomnia symptoms indirectly by the mediation of anxiety and depression [53]. Social support as an external effect may be too weak to transform internal emotion and then to moderate the association between IU and insomnia directly. In addition, insomnia is highly related to the sleep environment, which should ideally be a peaceful and quiet bedroom. Hence, the moderation effect of social support may be limited.

The current research has several practical limitations. First, it is difficult to deduce causal inferences between variables and find the implicated changes of variables scores over time using a cross-sectional study. A longitudinal study could be performed to probe the cause and effect in future analogous studies. Second, convenient sampling was adopted to collect data, due to the rigorous closed-off management enforced in universities and the inability to obtain a sampling frame of all college students in Wuhan, which could not represent interest population and exit bias. Third, all the scales retrieved in this study were self-submitted by participants. Hence, this result may have underlying prejudice. Nonetheless, all similar scales have been utilized in the previous study [17,44]. Accordingly, further study could be carried out among different groups to explore the relationship between IU, social support, anxiety, depression and insomnia. These subjects need serious qualitative surveys including in-depth interviews.

## 5. Conclusions

This study has significant implications to intervene in the mental health of college students with extreme uncertainty of COVID-19. The hypothesis has been verified in this research, except for insomnia. On the one hand, COVID-19-related intolerance of uncertainty was positively associated with adverse mental health outcomes (anxiety, depression, and insomnia), and social support was negatively associated with adverse mental health outcomes. In addition, back-to-school students who are certain and uncertain that COVID-19 will rebound again were significantly more anxious and depressed than those with optimistic attitudes. Government departments should pay high attention to the mental health problems evoked by intolerance of uncertainty. On the other hand, our study highlighted that social support as a moderator could buffer the relationship between IU and mental health, including anxiety and depression during unprecedentedly uncertain times. While, this research fails to prove the moderation effect of social support on the relationship between IU and insomnia. Further research can be carried out to explore the internal mechanism of insomnia. In light of the current literature, no study has explored the moderation of social support between IU and mental health problems among college students during the COVID-19 era. Based on the results above, low COVID-19-related uncertainty and strong social support from friends, teachers, and family members can eliminate mental health problems effectively.

## Figures and Tables

**Table 1:** Demographics information.

Demographic	N	%
Sex		
Male	475	46.7
Female	542	53.3
Age		
18–21	431	42.4
22–25	513	50.4
26–29	63	6.2
30 and above	10	1.0
Highest Education level		
Undergraduate	490	48.2
Master’s degree	447	44.0
Doctorate and above	80	7.9
Department category of the university		
Department of Medical	363	35.7
Non-Medical	654	64.3
How long do you expect to graduate		
Within 1 year	348	34.2
Within 2 years	336	33.0
Within 3 years	242	23.8
4 years and longer	91	8.9
Whether the COVID-19 epidemic in China will rebound again		
Yes	322	31.7
No	258	25.4
Uncertain	437	43.0

**Table 2:** Descriptive Statistics and Pearson’s Correlations among Variables.

Variable	M	SD	1	2	3	4	5
1.IU-12	36.01	7.379	1 **				
2.GAD-7	11.36	4.297	0.43 **	1 **			
3.PHQ-9	14.18	5.180	0.43 **	0.75 **	1 **		
4.ISI-7	13.76	5.495	0.38 **	0.53 **	0.64 **	1 **	
5.SSQ	29.18	9.521	−0.13 **	−0.28 **	−0.30 **	−0.28**	1 **

Note. IU-12 = 12-item Intolerance of Uncertainty Scale, GAD-7 = 7-item General Anxiety Disorder Scale, PHQ-9 = 9-item Patient Health Questionnaire, ISI-7 = 7-item Insomnia Severity Index Scale, SSQ = Social Support Questionnaire. ** *p* < 0.001.

**Table 3:** One-way Multivariate Analysis of Variance.

Variables	Group 1		Group 2		Group 3		Total		F	P
	M	SD	M	SD	M	SD	M	SD		
GAD-7	12.12	4.334	10.86	4.194	11.10	4.264	11.36	4.297	7.721	0.000
PHQ-9	14.90	5.382	13.65	5.119	13.96	5.017	14.18	5.180	4.868	0.008
ISI-7	14.28	5.770	13.87	5.432	13.32	5.297	13.76	5.495	2.855	0.058

Note. Group 1 = COVID-19 epidemic in China will rebound again; Group 2 = COVID-19 epidemic in China will not rebound again; Group 3 = Uncertain whether COVID-19 epidemic in China will rebound again.

**Table 4:** Regression Results Using Process in SPSS.

Variable	Anxiety			Depression			Insomnia		
	β	S.E.	t	β	S.E.	t	β	S.E.	t
IU	0.40	0.03	14.27 **	0.39	0.03	14.05 **	0.34	0.03	12.00 **
Social support	−0.23	0.03	−8.38 **	−0.26	0.03	−9.26 **	−0.25	0.03	−8.78 **
IU *social support	−0.07	0.03	−2.73 **	−0.06	0.03	−2.45 *	−0.04	0.03	−1.61
R^2^	0.25			0.26			0.24		
F	42.23 **			44.94 **			38.82 **		

Note. S.E., standard error of mean. ** *p* < 0.001, * *p* < 0.05.

**Table 5:** Moderation Effect of Social Support.

Variable	Anxiety			Depression		
	β	S.E.	t	β	S.E.	t
Zero social support	0.40	0.03	14.27 **	0.39	0.03	14.05 **
High social support(+1SD)	0.33	0.04	8.54 **	0.33	0.04	8.56 **
Low social support(-1SD)	0.47	0.04	12.62 **	0.45	0.04	12.26 **

Note. S.E., standard error of mean. ** *p* < 0.001.

## Data Availability

The data presented in this study are available on request from the corresponding author. The data are not publicly available due to privacy.
